# Minimally invasive video-assisted thyroidectomy for the early-stage differential thyroid carcinoma

**DOI:** 10.1186/1479-5876-10-S1-S13

**Published:** 2012-09-19

**Authors:** Jian-jun Yu, Shan-lin Bao, Sheng-lin Yu, Da-Qing Zhang, Wings TY Loo, Louis WC Chow, Li Su, Zhen Cui, Kai Chen, Li-Qiong Ma, Ning Zhang, Hui Yu, Yun-Zhen Yang, Yu Dong, Adrian YS Yip, Elizabeth LY Ng

**Affiliations:** 1 Department of Surgical-oncology, Affiliated Tumor Hospital, Ningxia Medical University, Ningxia, PRC; 2 Department of Breast and Mini-invasive Surgery, Ningxia People's Hospital, Ningxia, PRC; 3 UNIMED Medical Institute and Organisation for Oncology and Translational Research, Hong Kong SAR; 4 Department of Clinical Oncology, Ningxia Peoples’ Hospital, Ningxia, PRC; 5 Department of Surgical-oncology, the Affiliated Hospital, Bengbu Medical College, Bengbu, PRC; 6 Department of Pathology, the Affiliated Hospital, Ningxia Medical University, Ningxia, PRC; 7 Medical College of Xi’an Jiaotong University, Xi’an, PRC

## Abstract

**Background:**

Minimally invasive video-assisted thyroidectomy (MIVAT), the modified Miccoli’s thyroid surgery, is the most widespread minimally invasive technique and has been widely used for treatment of thyroid disease. This study aimed to verify the potential benefits of the modified Miccoli’s thyroid surgery, determine the feasibility of the MIVAT for early-stage differential thyroid carcinoma and evaluate the likelihood of the surgical method as a standard operation for early malignant thyroid carcinoma.

**Methods:**

A total of 135 patients were retrospectively compared which included two groups of patients: the first group underwent the conventional thyroidectomy; the other group underwent MIVAT. Patients with thyroid nodule smaller than 20 mm and without previous neck surgery were included while those with wide-ranging and distant metastases of cervical tissues, or any suspected thyroid nodal metastases were excluded for analysis. MIVAT and the central compartment (level VI) lymph nodes dissection (LND) were considered as a new treatment method for this retrospective study. In addition to the comparison of surgical outcomes between the new treatment and the conventional thyroid surgery, other surgical parameters including operative time, operative volume of hemorrhage, incisional length, postoperative volume of drainage, length of hospitalization, accidence of hoarse voice, accidence of bucking, accidence of hypocalcemia and peak angle of cervical axial rotation were also compared.

**Results:**

Out of 135 patients, 111 patients underwent conventional thyroid surgery and 24 patients underwent MIVAT plus level VI LND for treatment of early-stage differential malignant carcinoma. Patients who received the new surgical treatment had significantly shorter incisional length (3.1 cm *vs.* 6.9 cm, p < 0.0001), shorter operative time (109 min *vs*. 139 min, p = 0.014) and fewer operative hemorrhage (29.5 ml *vs.* 69.7 ml, p < 0.0001) when compared to the conventional treatment. Postoperative peak angle of cervical axial rotation of patients treated with MIVAT was less than those treated with conventional surgery (L: 31.5° *vs.* 39.0°, p < 0.0001; R: 31.5° *vs.* 38.0°, p < 0.0001). Incisional wound infection, postoperative hoarse voice, bucking and hypocalcemia were not observed in all patients. Postoperative analgetica was not required as well.

**Conclusions:**

Compared with conventional thyroid surgery for early-stage differential thyroid carcinoma, the new surgical treatment could be considered as an alternative surgical method for treatment of early-stage thyroid carcinoma since it was feasible, safe and clinically effective with better surgical and cosmetic outcomes.

## Background

Conventional thyroid surgery has been widely used for treatment of malignant thyroid disease with a significant reduction of mortality since the 19th century [1]. Although conventional thyroidectomy is deemd the “standard procedure” which is effective, well-tolerated, and safe, it requires transverse incisions through the skin of the neck and usually leaves a 6 to 8 centimeters neck scar [2]. An unsightly scar on the anterior neck could be distressing for both the patients and the surgeons. Traditional open thyroidectomy is performed through a midline cervical incision that can be associated with parasthesias, hypethesias, and unsightly scarring. Additionally, voice changes and dysphagia without discrete clinical findings affect patients postoperatively [3]. The evolution of surgical therapy has therefore entered the period of improved cosmetic outcomes and better postoperative recovery.

Minimally invasive surgery has been proposed recently for the management of thyroid disorders [4,5] and has gradually been agreed to treat early-stage disease [6], Minimally invasive video-assisted thyroidectomy (MIVAT), previously adopted for treatment of benign thyroid disease [7-9], is one of the procedures of minimally invasive thyroid surgery and has been performed on patients with malignant disease since 2002 [4,10]. MIVAT is a reproducible technique without gas insufflation and it involves a 1.5–2.0-cm incision 2 cm above the sterna notch, strap muscle dissection, and endoscope insertion [11]. Cosmetic improvements and reduced postoperative pain, length of hospital stay and social costs are some of the major advantages of the minimally invasive surgery [8,12-16]. The esthetic benefits is important especially for young patients. However, MIVAT does not provide as much operative space as the conventional one for CO_2_-insufflation, but a better operative space could be created by using a new modified retractor. The modified retractor and ultrasound knife have modified thyroid surgery successfully with a significant improvement in not only operative fields, but also postoperative outcomes. The feasible and safe technique offers similar complication rates to conventional thyroidectomy, but with better cosmetic outcomes and less postoperative distress [6,17,18]

In this paper, we retrospectively compared the results obtained in patients who underwent MIVAT plus the central compartment (level VI) lymph nodes dissection (LND) to those underwent conventional thyroidectomy (CT) to testify the feasbility, safety and cosmetic advantages of the new minimally invasive surgery technique for treatment of the early-stage differential thyroid carcinoma. Several surgical parameters including operative time, length of incision, operative hemorrhage, postoperative drainage, length of hospitalization and peak angles of cervical axial rotation were investigated.

## Methods

### Patients and assessments

Between 1st June 2009 and 1st April 2011, 135 consecutive patients diagnosed with early-stage differential thyroid carcinoma with the largest diameter of less than 20 mm were treated at the Department of Oncological Surgery, the Affiliated Cancer Hospital of Ningxia Medical University. No specific exclusion criteria were used for this retrospective study. Patients were divided into two groups according to the types of thyroid surgery received. Patients who underwent CT and MIVAT plus level VI LND were classified as CT group and MIVAT group respectively. Preoperative assessments including thyroid ultrasound, thyroid function tests, and methoxyisobutylisonitrile scan were performed for all cases. Gross pathological examinations during operations and paraffin pathological examinations after operations were conducted.

### Surgical techniques

**Figure 1:**
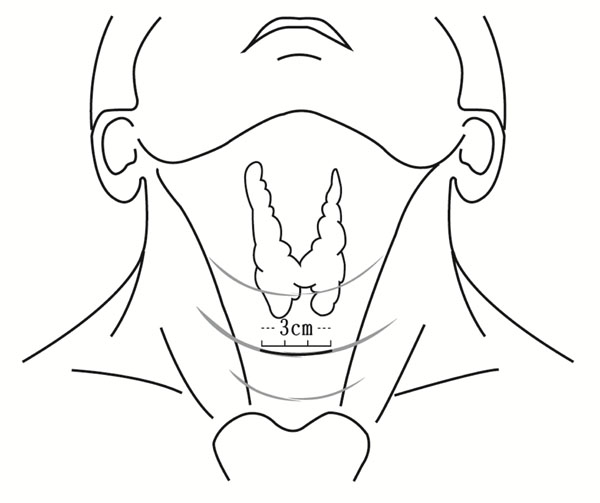
Illustration of the incision design for the minimally invasive video-assisted thyroidectomy

**Figure 2:**
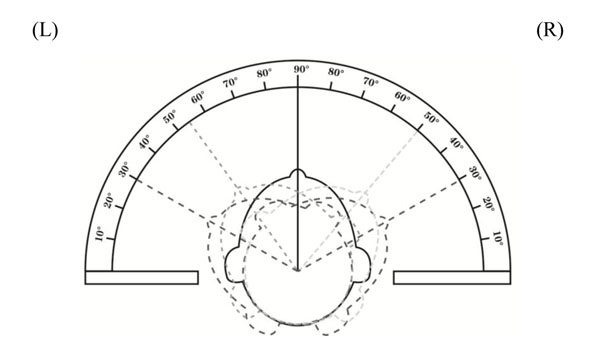
**Illustration of the measurement of peak angle of cervical axial rotation** The weight line means preoperative measurement and the light line means postoperative measurement. Left (L) and right (R) peak angles of axial rotation were measured. Postoperative angles were compared between groups.

All patients had surgery performed in the supine position under general anesthesia. The surgical procedure for MIVAT was described in elsewhere [4,7]. In our study, the operative incisions of MIVAT were made in the last cervical wrinkle with the length of operative incision of approximately 2.5 cm, but not that about 3.0 cm from suprasternal notch as usual (Figure 1). The skin, subcutaneous tissue and platysma was cut in order. The skin flap was separated from cricoid cartilage to superior fovea of sternum and the linea alba was opened. The lobus glandulae thyroideae was exposed completely using a modified retractor which was suspended and fixed. The 30-degree endoscope was then inserted into the working space. Using the amplified dynamic graphs, we could easily differentiate between relative vessels and nerves, and excise superior and inferior thyroid artery, middle thyroid veins and the tumors by the ultrasound scalpel. After dissection, the residual thyroid, platysma, subcutaneous tissue and skin was closed in order and drainage tubes were inserted. In our study, MIVAT and the level VI LND was selected as the new surgical treatment method. All surgical parameters including length of incision, operative time, and operative hemorrhage were recorded. At the second day after the operation, the peak angle of cervical axial rotation was measured for both left and right sides (Figure 2). Postoperative drainage and the length of hospitalization were also recorded. All parameters were compared between groups.

### Statistical analysis

The non-parametric Mann-Whitney test was used to compare the means of parameters between two groups. A P value of less than 0.05 was considered statistically significant. Statistical calculations were performed using SPSS version 14.0 for Windows.

## Results

### Patient characteristics

A total of 135 patients were included for this retrospective study and among which, 111 patients underwent CT, and 24 patients underwent MIVAT plus level VI LND which were assigned as CT group and MIVAT group respectively. The clinical characteristics did not show statistical significance between groups (Table 1).

**Table 1:** Clinical characteristics of subjects

Clinical characteristics	MIVAT group	CT group	P value
	(n = 24)	(n = 111)	
Age (years)			
Mean ± SD Range	45 ± 10 23 – 60	47 ± 11 23 – 73	0.542
Size of tumors (cm)			
Mean ± SD Range	1.1 ± 0.6 0.2 – 2.0	1.2 ± 0.5 0.2 – 2.0	0.267

Abbreviations: CT: conventional thyroidectomy; MIVAT: Minimally invasive video-assisted thyroidectomy; SD: standard deviation

### Surgical treatment

Surgical details of both groups were summarized in table 2. Significant difference was observed in a number of surgical parameters. The average incision length was 3.8 cm shorter in MIVAT group than that in CT group (3.1 cm ± 1.1 cm *vs* 6.9 cm ± 1.3 cm, p < 0.0001). The mean operative time, the duration between skin incision and closure, was also 30 minutes (min) shorter in MIVAT group than that in CT group (109 min ± 49 min *vs* 139 min ± 66 min, p = 0.014). Operative hemorrhage was significantly lower in volume in MIVAT group than that in CT group, but no significant difference was observed in postoperative drainage. During operation, patients in CT group on average had approximate 40.2 ml more blood loss than that in MIVAT group (69.7 ml ± 52.7 ml *vs* 29.5 ml ± 43.1 ml, p < 0.0001).

**Table 2:** Surgical treatment details of subjects

Surgical parameters	MIVAT group	CT group	P value
	(n = 24)	(n = 111)	
Operative time (minutes)	109 ± 49	139 ± 66	0.014*
Length of incision (cm)	3.1 ± 1.1	6.9 ± 1.3	<0.0001*
Operative hemorrhage (mL)	29.5 ± 43.1	69.7 ± 52.7	<0.0001*
Postoperative drainage (mL)	42.9 ± 28.9	53.4 ± 49.1	0.729
Length of hospitalization (days)	4.7 ± 1.9	5.5 ± 3.1	0.548
Peak angle of cervical axial rotation (degree)			
Left	31.5 ± 3.5	39.0 ± 4.1	<0.0001*

### Surgical outcomes

None of patients from both groups required analgetica and experienced incisional infection after surgery. Postoperative hoarse voice, bucking and hypocalcemia were not observed in all patients as well. After 3-month postoperative follow-up, all patients were still disease-free. Compared with patients in CT group, patients from MIVAT group had satisfactory wound healing and good cosmetic outcomes. Significant difference in the extent of surgical injury as indicated by the peak angle of left (L) and right (R) cervical axial rotation were observed between MIVAT and CT groups (L: 31.5° ± 3.5°*vs* 39.0° ± 4.1°, p < 0.0001; R: 31.5°± 3.2° *vs* 38.0° ± 3.2°, p < 0.0001) two days after the operation.

## Discussion

The first MIVAT was performed in the 1990s and reported [1]. The new surgical procedure has made significant evolution in the endoscopic thyroid surgery [19-24] and offered better healing and cosmetic outcomes compared to conventional open thyroidectomy in thyroid diseases [6,17]. Recent research has also demonstrated the feasibility of using MIVAT for treatment of early-stage thyroid carcinoma with fewer postoperative complications and better cosmetic outcomes, albeit similar prognosis [25,26].

In our records, young female patients often had conspicuous scars on cadavers after CT. A previous report has demonstrated that thyroid gland manipulation did not differ between video-assisted thyroidectomy and conventional surgery, and video-assisted thyroidectomy did not confer additional risk of thyroid capsule rupture and cell seeding [27]. However, we could still find some patients who received MIVAT developed small but conspicuous scars in our initial cases. Through relative researches about anatomy and cosmetology, it was speculated that the natural cervical wrinkles could hide incisions and postoperative scarring to achieve perfect appearances.

MIVAT is widely accepted for treatment of different thyroid diseases including benign thyroid diseases [6] and yet to be adopted for treatment of low-risk or early-stage malignant thyroid diseases for fear of incomplete surgical removal of tumor and metastatic lymph nodes. Lai et al. [28] reported clear surgical margins of resection for all patients with malignant thyroid lesions with a comparable postoperative radioiodine uptake in thyroid bed (median 1.76%; range 0.21% – 3.02%) to open thyroidectomy in the previous report [9]. The expansion of oncologic indications for MIVAT did not compromise patient’s safety and rate of complication. More evidence has now demonstrated that MIVAT can reduce the duration of postoperative stay, minimize the postoperative distress and better cosmetic outcomes [25,26,29].

In our study, postoperative assessment of the peak angle of cervical axial rotation was performed to investigate the degree of surgical injury. Patients with larger angle of axial rotation indicated less injury to the neck. The results, showing a lesser extent of injury to the neck with the significantly larger left and right axial rotation angles in patients treated with MIVAT than CT, has further demonstrated the surgical potential of using the minimally invasive surgery to treat patients with early malignant thyroid carcinoma. Notwithstanding the better cosmetic outcome and less postoperative distress, it is important to further investigate the survival benefit of the minimally invasive surgical therapy and determine the therapeutic paradigm for patients with early-stage thyroid cancer. Despite insignificant difference in survival outcomes between patients treated with MIVAT and CT [26], another question about which group of patients best selected for the minimally invasive surgery will come up and therefore further research is still warranted.

## Conclusions

The new minimally invasive thyroid surgery, MIVAT plus level VI LND, is feasible and safe for treatment of early-stage thyroid carcinoma. The cosmetic outcomes and surgical outcomes are better than conventional thyroid surgery. Further investigation is warranted to investigate the possibility to replace open thyroidectomy in a particular group of patients with early-stage thyroid carcinoma.

## Competing interests

The author(s) declare that they have no competing interests.

## Authors' contributions

HY, ZC, ZM, CL, XY, LS, TM, HY, YY, DZ, KC, YD, JY, SB, and SY carried out the scientific research. JY, WTYL, LWCC, AYSY and ELYN participated in the writing of the manuscript. WTYL and AYSY performed statistical analysis. JY and LWCC provided expert opinion for the study. JY, SB, and SY were involved in consultation for the research. JY was the initiator of the study.
